# In vitro piperaquine susceptibility is not associated with the Plasmodium falciparum chloroquine resistance transporter gene

**DOI:** 10.1186/1475-2875-12-431

**Published:** 2013-11-25

**Authors:** Aurélie Pascual, Marilyn Madamet, Lionel Bertaux, Rémy Amalvict, Nicolas Benoit, Dominique Travers, Julien Cren, Nicolas Taudon, Christophe Rogier, Daniel Parzy, Bruno Pradines

**Affiliations:** 1 Département d’Infectiologie de Terrain, Unité de Parasitologie, Institut de Recherche Biomédicale des Armées, Marseille, France; 2 Aix Marseille Université, Unité de Recherche sur les Maladies Infectieuses et Tropicales Emergentes, UM 63, CNRS 7278, IRD 198, Inserm 1095, Marseille, France; 3 Centre National de Référence du Paludisme, Marseille, France; 4 UMR MD3, Aix Marseille Université, Institut de Recherche Biomédicale des Armées, Marseille, France; 5 Equipe Résidente de Recherche en Infectiologie Tropicale, Institut de Recherche Biomédicale des Armées, Hôpital d’Instruction des Armées Laveran, Marseille, France; 6 Institut Pasteur de Madagascar, Antananarivo, Madagascar

**Keywords:** Malaria, *Plasmodium falciparum*, Anti-malarial, Resistance, *in vitro*, Molecular marker, Piperaquine, Chloroquine, *pfcrt*

## Abstract

**Background:**

Dihydroartemisinin-piperaquine is a new ACT that is administered as single daily dose for three days and has been demonstrated to be tolerated and highly effective for the treatment of uncomplicated *Plasmodium falciparum* malaria. Piperaquine was used alone to replace chloroquine as the first-line treatment for uncomplicated malaria in China in response to increasing chloroquine resistance in the 1970s. However, the rapid emergence of piperaquine-resistant strains that resulted in the cessation of its use in China in the 1980s, suggests that there is cross-resistance between piperaquine and chloroquine. Very few data are available on cross-resistance between piperaquine and chloroquine, and the data that do exist are often contradictory.

**Methods:**

In total, 280 *P. falciparum* isolates, collected between April 2008 and June 2012 from patients hospitalized in France with imported malaria from a malaria-endemic country, were assessed *ex vivo* for piperaquine and chloroquine susceptibilities by using the standard 42-hour 3H-hypoxanthine uptake inhibition method. The chloroquine resistance-associated mutation K76T in *pfcrt* was also investigated for the 280 isolates.

**Results:**

The IC_50_ for piperaquine ranged from 9.8 nM to 217.3 nM (mean = 81.3 nM. The IC_50_ for chloroquine ranged from 5.0 nM to 1,918 nM (mean = 83.6 nM. A significant but low correlation was observed between the Log IC_50_ values for piperaquine and chloroquine (r = 0.145, p < 0.001). However, the coefficient of determination of 0.021 indicates that only 2.1% of the variation in the response to piperaquine is explained by the variation in the response to chloroquine. The mean value for piperaquine was 74.0 nM in the *Pfcrt* K76 wild-type group (no = 125) and 87.7 nM in the 76 T mutant group (no = 155). This difference was not significant (p = 0.875, Mann Whitney U test).

**Conclusions:**

The present work demonstrates that there was no cross-resistance between piperaquine and chloroquine among 280 *P. falciparum* isolates and that piperaquine susceptibility is not associated with *pfcrt*, the gene involved in chloroquine resistance. These results confirm the efficacy of piperaquine in association with dihydroartemisinin and support its use in areas in which parasites are resistant to chloroquine.

## Background

Over the past 20 years, many strains of *Plasmodium falciparum* have become resistant to chloroquine and other anti-malarial drugs [1]. In 2002, the World Health Organization (WHO) recommended that artemisinin-based combination therapy (ACT) be used to treat all cases of uncomplicated malaria. Different formulations of ACT have been evaluated: artesunate-sulphadoxine-pyrimethamine, artesunate-amodiaquine, artemether-lumefantrine, artesunate-mefloquine, artesunate-chlorproguanil-dapsone, artesunate-pyronaridine and, more recently, dihydroartemisinin-piperaquine. Most of these formulations are available as fixed-dose co-formulations, which are convenient, facilitate improved adherence and help prevent misuse.

Dihydroartemisinin-piperaquine (Artekin^®^, Duo-Cotecxin^®^, Eurartesim^®^) is a new ACT that is administered as single daily dose for three days and has been demonstrated to be tolerated and highly effective for the treatment of uncomplicated malaria in Asia [2,3] and the treatment of uncomplicated *P. falciparum* malaria in Africa [4,5]. Dihydroartemisinin-piperaquine seems to have a better post-treatment prophylactic effect than artemether-lumefantrine [6-8] or artesunate-amodiaquine [9]. Since 2012, dihydroartemisinin-piperaquine has been available for the treatment of uncomplicated malaria in France.

Piperaquine, a bisquinoline, was used alone to replace chloroquine as the first-line treatment for uncomplicated malaria in China in response to increasing chloroquine resistance in the 1970s. However, the rapid emergence of piperaquine-resistant strains resulted in the cessation of its use in China in the 1980s [10].

This rapid emergence of piperaquine-resistant strains suggests that there is cross-resistance between piperaquine and chloroquine. Very few data are available on cross-resistance between piperaquine and chloroquine, and the data that do exist are often contradictory. A positive significant correlation was found for 63 isolates from the China-Myanmar border area (r = 0.79, p < 0.0001) [11], 54 isolates from Papua New Guinea (r = 0.51, p < 0.001) [12] and 103 isolates from Cameroon (r = 0.257, p < 0.05) [13], whereas no significant correlation was observed for 199 isolates from Uganda (r = 0.121, p = 0.15) [14], 115 culture-adapted isolates from Kenya (r = 0.16, p = 0.13) [15], 23 strains from 16 different countries (r = 0.199, p = 0.366) [16] or 181 isolates of imported malaria from 19 countries (r = 0.036, p = 0.634) [17]. In addition, very few data are available on the association between piperaquine susceptibility and polymorphisms in the gene involved in chloroquine resistance, *pfcrt* (*P. falciparum* chloroquine resistance transporter) [18].

The objectives of the present work were to evaluate the cross-resistance between piperaquine and chloroquine in 280 fresh isolates of *P. falciparum* and to investigate the association between piperaquine and chloroquine susceptibility and the K76T mutation in *pfcrt*.

## Methods

### Patients and sample collection

In total, 280 *P. falciparum* isolates were collected between April 2008 and June 2012 from patients hospitalized in France with imported malaria from a malaria-endemic country (Angola, Benin, Burkina Faso, Cameroon, Central African Republic, Chad, Comoros, Congo, Ivory Coast, Gabon, Gambia, Ghana, Guinea, India, Madagascar, Mali, Mauritania, Mozambique, Niger, Senegal, Thailand, Togo, Zambia). Informed consent was not required for this study because the sampling procedures and testing are part of the French national recommendations for the care and surveillance of malaria. Venous blood samples were collected in Vacutainer^^®^^ ACD tubes (Becton Dickinson, Rutherford, NJ, USA) before treatment and were transported at 4°C from French hospitals located in Aix en Provence, Bordeaux, Chambery, Frejus, Grenoble, Lyon, Marseille, Metz, Montpellier, Nice, Nimes, Pau, Toulon, Toulouse, and Valence to the Institute of Biomedical Research of the French Army (IRBA) in Marseille within 72 hours of collection. The Case Report Form was provided at the same time, either as a paper copy or electronically.

Thin blood smears were stained using a RAL^^®^^ kit (Réactifs RAL, Paris, France) and were examined to determine *P. falciparum* density and confirm mono-infection. Parasitized erythrocytes were washed three times with RPMI 1640 medium (Invitrogen, Paisley, UK) buffered with 25 mM HEPES and 25 mM NaHCO_3_. If parasitaemia exceeded 0.5%, infected erythrocytes were diluted to 0.5% with uninfected erythrocytes (human blood type A+) and re-suspended in RPMI 1640 medium supplemented with 10% human serum (Abcys S.A. Paris, France), for a final haematocrit of 1.5%. The susceptibility of the 280 isolates was assessed without culture adaptation.

### Drugs

Piperaquine was obtained from Shin Poong Pharm Co. (Seoul, Korea) and was dissolved first in methanol and then diluted in water to obtain final concentration ranging from 0.8 to 1,000 nM. Chloroquine was purchased from Sigma (Saint Louis, MO, USA) and was dissolved first in methanol and then diluted in water to final concentrations ranging from 5 nM to 3,200 nM. Batches of plates were tested and validated using the chloroquine-susceptible 3D7 strain (West Africa) and the chloroquine-resistant W2 strain (Indochina) (MR4, Virginia, USA) in three to six independent experiments using the conditions described in the paragraph below. The two strains were synchronized twice with sorbitol before use [19], and clonality was verified every 15 days using PCR genotyping of the polymorphic genetic markers *msp1* and *msp2* and using microsatellite loci [20,21] and additionally verified each year by an independent laboratory from the Worldwide Anti-malarial Resistance Network (WWARN).

### Ex vivo assay

For *ex vivo* isotopic microtests, 200 μl/well of the suspension of synchronous parasitized red blood cells (final parasitaemia, 0.5%; final haematocrit, 1.5%) were distributed in 96-well plates pre-dosed with anti-malarial drugs. Parasite growth was assessed by adding 1 μCi of tritiated hypoxanthine with a specific activity of 14.1 Ci/mmol (Perkin-Elmer, Courtaboeuf, France) to each well at time zero. The plates were then incubated for 42 hours in controlled atmospheric conditions that consisted of 10% O_2_, 5% CO_2_, and 85% N_2_ at 37°C with a humidity of 95%. Immediately after incubation, plates were frozen and then thawed to lyse erythrocytes. The content of each well was collected on standard filter microplates (Unifilter GF/B; Perkin-Elmer) and washed using a cell harvester (Filter-Mate Cell Harvester; Perkin-Elmer). Filter microplates were dried, and 25 μl of scintillation cocktail (Microscint O; Perkin-Elmer) was placed in each well. Radioactivity incorporated in nucleotides by the parasites was measured with a scintillation counter (Top Count; Perkin-Elmer).

The drug concentration able to inhibit 50% of parasite growth (IC_50_) was assessed by the drug concentration corresponding to 50% of the incorporation of tritiated hypoxanthine by the parasite in the drug-free control wells. The IC_50_ value was determined by non-linear regression analysis of log-based dose–response curves (Riasmart, Packard, Meriden, USA).

### Nucleic acid extraction

Total genomic DNA of each strain was isolated using the QIAamp^®^ DNA Mini kit according to the manufacturer’s recommendations (Qiagen, Germany).

### Pfcrt single-nucleotide polymorphisms (SNPs)

A 546-nucleotide fragment of the *Pfcrt* gene (containing codon 76) was amplified by PCR using CRTP1-sense 5′-CCG TTA ATA ATA AAT ACA CGC AG-3′ and CRTP1-antisense 5′-CGG ATG TTA CAA AAC TAT AGT TAC C-3′ primers [22]. The reaction mixture for PCR amplifications included 2.5 μl of genomic DNA, 2.5 μl of 10X reaction buffer (Eurogentec), 0.5 μM of each primer, 200 μM of a deoxynucleoside triphosphate mixture (dGTP, dATP, dTTP and dCTP) (Euromedex, Souffelweyersheim, France), 2.5 mM MgCl_2_ and 1 unit of RedGoldStar^®^ DNA polymerase (Eurogentec) in a final volume of 25 μl. The thermal cycler (T3 Biometra, Archamps, France) was programmed as follows: an initial 94°C incubation for 5 min, 40 cycles of 94°C for 20 sec, 56°C for 20 sec, 60°C for 40 sec, and a final 5-min extension step at 60°C. The PCR products were loaded on a 1.5% agarose gel containing 0.5 μg/mL ethidium bromide. The PCR products were diluted 1:100 in distilled water, and 2.5 μl of the final dilution was used for the second PCR. This PCR amplified a 275 bp segment around the mutation using a common inner primer CRTP3-sense 5′-TGA CGA GCG TTA TAG AG-3′ coupled with either CRTP4m-antisense 5′-GTT CTT TTA GCA AAA ATT G-3′ (detects the 76 T codon) or CRTP4w-antisense 5′-GTT CTT TTA GCA AAA ATT T-3′ (detects the 76 K codon). The reaction mixture for the PCR amplifications included 2.5 μl of diluted PCR product, 2.5 μl of 10X reaction buffer (Eurogentec), 0.5 μM of each primer, 200 μM deoxynucleoside triphosphate mixture (dGTP, dATP, dTTP and dCTP) (Euromedex, Souffelweyersheim, France), 1.5 mM MgCl_2_ and 0.75 U of RedGoldStar^®^ DNA polymerase (Eurogentec) in a final volume of 25 μl.

The PCR conditions were at 94°C for 5 min, 15 cycles at 94°C for 20 sec, 48.5°C for 20 sec, 64°C for 40 sec, and a final 5-min extension step at 64°C. Purified genomic DNA from *P. falciparum* clones 3D7 (chloroquine susceptible) and W2 (chloroquine resistant) were used as positive controls, and water and human DNA were used as negative controls. The PCR products from the amplification reactions were evaluated by electrophoresis on 2% agarose gels.

### Statistical analysis

Data were analysed using R software (version 2.10.1). Assessment of standard anti-malarial drugs cross-resistance between piperaquine and chloroquine drugs was measured by pairwise correlation of IC_50_ values of all isolates and estimated by coefficient of correlation of Pearson (*r*) and coefficient of determination (*r*^*2*^). Differences between the chloroquine and piperaquine IC_50_ values of isolates and *Pfcrt* K76T were compared using the Mann Whitney U test.

## Results

**Figure 1:**
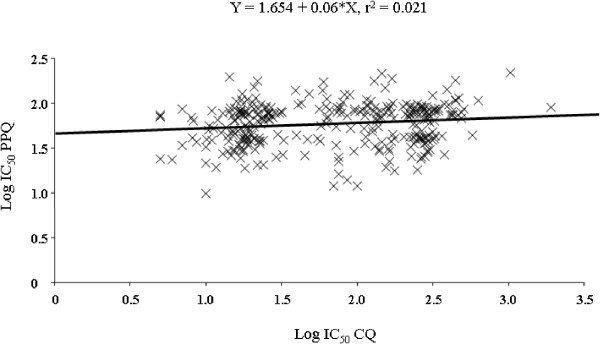
**Pearson’s correlation analysis of the Log IC**_**50 **_**values of piperaquine and chloroquine.**

The IC_50_ for piperaquine ranged from 9.8 nM to 217.3 nM (mean = 81.3 nM; 95% confidence interval 71.3-92.7). The IC_50_ for chloroquine ranged from 5.0 nM to 1918 nM (mean = 83.6 nM; 95% confidence interval 71.0-98.3). Fifty three% of the isolates showed IC_50_ > 100 nM for chloroquine. A significant correlation was observed between the Log IC_50_ values for piperaquine and chloroquine (r = 0.145, p < 0.001) (Figure 1).

Of the 280 isolates, 125 were wild type (K76), and 155 were mutated (76T). The mean value for chloroquine IC_50_ was 31.3 nM (95% CI 25.4-38.7) in the wild-type group and 184.5 (95% CI 157.4-215.8) in the mutant group. This difference was significant (p = 0.001, Mann Whitney U test). The mean value for piperaquine was 74.0 nM (95% CI 62.9-87.0) in the wild-type group and 87.7 (95% CI 71.9-106.9) in the mutant group. This difference was not significant (p = 0.875, Mann Whitney U test).

## Discussion

The IC_50_ for piperaquine ranged from 9.8 nM to 217.3 nM (mean = 81.3 nM; 95% confidence interval 71.3-92.7). These values are greater than the geometric means for isolates from Cameroon (geometric mean = 39 nM) [13], the Thai-Burmese border (49 nM) [23], Kenya (50 nM) [15], Uganda (6.1 nM) [14], Indonesia (21.8 nM) [24], and Papua New Guinea [12]. The comparison of IC_50_s across studies is likely hampered by different methodology in assessing these. The isolate with the highest IC_50_ for piperaquine (217.3 nM) was also resistant to chloroquine (1,029 nM). There is no consensus threshold indicating piperaquine in vitro resistance or reduced susceptibility.

*In vitro* cross-resistance was assessed using the pairwise correlation of the Log IC_50_ values of the 280 isolates (Figure 1). A significant correlation was observed between the Log IC_50_ values for piperaquine and chloroquine (r = 0.145, p <0.001). However, this value is too low to suggest that there is cross-resistance between piperaquine and chloroquine. For a correlation to imply that two compounds share common mechanisms of action or resistance, which could induce cross-resistance, the coefficient of determination (r^2^) must be high. Here, the coefficient of determination of 0.021 indicates that only 2.1% of the variation in the response to piperaquine is explained by the variation in the response to chloroquine. These data are in accordance with the majority of the previous studies, which found weak coefficients of determination [13-17]. This result suggests that piperaquine and chloroquine do not share common mechanisms of resistance. However, positive significant correlation was found for 63 isolates from the China-Myanmar border area (r = 0.79, p < 0.0001) [11] and 54 isolates from Papua New Guinea (r = 0.51, p < 0.001) [12]. This difference in *in vitro* cross-resistance might be explained by the low sample numbers in these two studies and by geographical strain differences.

As expected, the 76T mutation is associated with chloroquine resistance (p = 0.001, Kruskal-Wallis test). The mean value for piperaquine was 74.0 nM (95% CI 62.9-87.0) in the wild-type group and 87.7 (95% CI 71.9-106.9) in the mutant group. This difference was not significant (p = 0.862, Kruskal-Wallis test). These data suggest that the 76T mutation is not associated with piperaquine-decreased susceptibility. These data are in accordance with previous data on 23 strains from 15 countries of Africa, Asia and South America [16] and 115 isolates from Kenya [15]. The absence of cross-resistance between piperaquine and chloroquine may be explained by the absence of an association between piperaquine resistance and *pfcrt.* The very weak correlation between piperaquine and chloroquine responses (only 2.1% of the variation in the response to piperaquine is explained by the variation in the response to chloroquine) could be explained by other polymorphisms involved in very minor way in chloroquine resistance, such as *pfmdr1* SNPs or copy number. Fieldwork has shown that the predictive value for chloroquine resistance and point mutations in the *pfmdr1* sequence resulting in amino acid changes varies depending on the geographic area [25,26]. Point mutations, most notably N86Y, have been associated with a decrease in the chloroquine susceptibility [27]. However, in some of these epidemiological studies, the number of chloroquine-susceptible samples is too limited to provide statistically meaningful analysis [26,28]. Using precautions, no or only weak relationships are established in *P. falciparum* between chloroquine resistance and mutations in *pfmdr1*[25]. However, previous works demonstrated that polymorphisms in *pfmdr1* gene or copy number are not associated with decreased susceptibility to piperaquine [11,15,16].

These field results are in contrast to experimental data that showed that genetically modified parasites with CVIET haplotypes had reduced susceptibility to piperaquine [29].

The present work demonstrated that piperaquine exhibits currently no cross-resistance with chloroquine in African *P. falciparum* isolates and that resistance to piperaquine is not associated with *pfcrt*, the gene involved in chloroquine resistance. The validity of this conclusion should be further supported by analysing more isolates, especially from South America and Asia. In addition, copy number variation of a chromosome 5 region, a genetic marker associated with high piperaquine IC_50_ in a piperaquine-selected *P. falciparum* line [30], should be evaluated for reduced *ex vivo* susceptibility. Nevertheless, these results confirm the efficacy of piperaquine in association with dihydroartemisinin and support its use in areas in which parasites are resistant to chloroquine.

## Competing interests

All authors declare that they have no competing interests.

## Authors’ contributions

MM, LB, NB, and JC carried out the molecular genetic studies. AP, RA and DT carried out the *ex vivo* evaluation of doxycycline susceptibility. The French National Reference Centre for Imported Malaria Study Group supervised, carried out and coordinated the field collections of patient isolates. NT, DP and BP conceived and coordinated the study. CR and BP analysed the data. AP, MM, NT, CR, DP, and BP drafted the manuscript. All the authors read and approved the final manuscript.

## Authors’ information

French National Reference Centre for Imported Malaria Study Group:

V Augis (CHU de Bordeaux, Bordeaux), D Basset (CHU Lapeyronnie, Montpellier), F Benoit-Vical (CHU de Rangueil, Toulouse), A Berry (CHU de Rangueil, Toulouse), N Bourgeois (CHU Caremeau, Nimes), F Conquere de Monbrison (CHU de Lyon, Lyon), P Delaunay (CHU de l’Archet, Nice), J Delmont (Hôpital Nord, Marseille), K Ezzedine (CHU de Bordeaux, Bordeaux), B Faugere (CHU La Timone, Marseille), T Gaillard (HIA Saint-Anne, Toulon), C Garabedian (CH du Pays d’Aix, Aix en Provence), D Malvy (CHU de Bordeaux, Bordeaux), P Marty (CHU de l’Archet, Nice), D Maubon (CHU de Grenoble 1, Grenoble), G Menard (HIA Saint-Anne, Toulon), P Millet (CHU de Bordeaux, Bordeaux), P Minodier (Hôpital Nord, Marseille), Montaut (CH de Pau, Pau), A Mottard (Hôpital de Fréjus-Saint Raphael, Fréjus), P Munier (CH de Valence, Valence), P Parola (Hôpital Nord, Marseille), S Picot (UMR 5246 CNRS, Lyon), T Pistone (CHU de Bordeaux, Bordeaux), C Pomares-Estran (CHU de l’Archet, Nice), J Puyhardy (HIA Legouest, Metz), D Raffenot (CH de Chambéry, Chambéry), M-C Receveur (CHU de Bordeaux, Bordeaux), H Savini (HIA Laveran, Marseille), F Simon (HIA Laveran, Marseille), S Vedy (HIA Legouest, Metz).
